# Mistreatment in an academic setting and medical students' perceptions about their course in São Paulo, Brazil: a cross-sectional study: Maus-tratos em um ambiente acadêmico e percepção sobre o curso entre estudantes de medicina em São Paulo, Brasil: um estudo transversal

**DOI:** 10.1590/1516-3180.2015.01332210

**Published:** 2015-03-17

**Authors:** Maria Fernanda Tourinho Peres, Fernanda Babler, Juliana Naomy Lacerda Arakaki, Irene Yamamoto do Vale Quaresma, Abraão Deyvid Alves de Lima Barreto, Andréa Tenório Correia da Silva, José Eluf-Neto

**Affiliations:** 1 MD, MSc, PhD. Professor, Department of Preventive Medicine, Faculdade de Medicina da Universidade de São Paulo (FMUSP), São Paulo, SP, Brazil.; 2 Medical Student, Undergraduate Research Training Program, Department of Preventive Medicine, Faculdade de Medicina da Universidade de São Paulo (FMUSP), São Paulo, SP, Brazil.; 3 MD, MSc. Doctoral Student, Postgraduate Program on Preventive Medicine, Department of Preventive Medicine, Faculdade de Medicina da Universidade de São Paulo (FMUSP), São Paulo, SP, Brazil.; 4 MD, MSc, PhD. Full Professor, Department of Preventive Medicine, Faculdade de Medicina da Universidade de São Paulo (FMUSP), São Paulo, SP, Brazil.

**Keywords:** Human rights abuses, Aggression, Social behavior, Bullying, Students, medical, Education, medical

## Abstract

**CONTEXT AND OBJECTIVE:**

High prevalence of mistreatment among medical students has been described in the worldwide literature since the 1980s. However, studies addressing the severity and recurrence of victimization and its effects on students' perceptions of their medical course are scarce. This study had the aim of estimating the prevalence of exposure to mistreatment that was considered to be severe and recurrent and its association with medical students' perceptions about their medical course.

**METHODS:**

A cross-sectional study was conducted in a medical school in São Paulo, Brazil. Three hundred and seventeen students from the first to the sixth year answered the online questionnaire.

**RESULTS:**

High prevalence of mistreatment during the course was found. Two thirds of the students considered the episodes to be severe, and around one third reported experiencing recurrent victimization. Occurences of mistreatment that the students considered to be severe were correlated with feeling overloaded and wanting to abandon the medical course.

**CONCLUSIONS:**

Occurrences of mistreatment within the academic environment are frequent in Brazil. The results suggest that mistreatment that was considered to be severe might negatively affect students' perceptions about their course.

## RESUMO

**CONTEXTO E OBJETIVO:**

Altas prevalências de maus-tratos entre estudantes de medicina vêm sendo descritas na literatura internacional desde a década de 1980. Estudos sobre a gravidade e recorrência da vitimização e seus efeitos na percepção dos alunos sobre o curso médico são escassos. Este estudo tem por objetivo estimar a prevalência de exposição a maus-tratos considerados graves e recorrentes e sua associação com a percepção dos estudantes de medicina sobre o curso médico.

**MÉTODOS:**

Estudo transversal realizado em uma escola médica em São Paulo, Brasil. Trezentos e dezessete estudantes do primeiro ao sexto ano responderam ao questionário *online* .

**RESULTADOS:**

Foram encontradas altas prevalências de maus-tratos durante o curso. Dois terços dos estudantes consideraram os maus-tratos graves e cerca de um terço referiu vitimização recorrente. A ocorrência de maus-tratos considerados graves pelos estudantes esteve associada a sentimento de sobrecarga e desejo de abandonar o curso médico.

**CONCLUSÃO:**

A ocorrência de maus-tratos no ambiente acadêmico é frequente no Brasil. Os resultados sugerem que os maus-tratos percebidos como graves podem afetar negativamente a percepção dos estudantes sobre o curso.

## INTRODUCTION

The high prevalence of mistreatment towards medical students has been identified as an important issue in medical education since the late 1980s and early 1990s. Since that time, studies conducted in different countries^1^^,^^2^^,^^3^^,^^4^^,^^5^^,^^6^^,^^7^^,^^8^^,^^9^^,^^10^^,^^11^^,^^12^^,^^13^ have corroborated the high prevalence of different forms of aggression within medical education, thus giving support to the idea that these abusive situations reflect a strongly hierarchical medical culture.^14^

Even though this problem was first described in the late 1980s, the prevalence of abuse, harassment and mistreatment among medical students remains high in different countries. Considering only studies that focused on the concept of bullying and thus limited their analysis to situations that were repetitive and persistent, the prevalence has ranged from 19.7% in Colombia^8^ to 52% in Pakistan.^6^ Studies that used broader concepts, including harassment, belittlement, discrimination and abuse not limited to persistent and chronic situations, have found higher prevalence rates: 18.9%,^4^ approximately 40%^2^^,^^5^ and more than 90%.^1^^,^^3^^,^^12^

Studies conducted in the US by Sheehan et al.^15^ and Rosenberg^16^ brought to light not only the high prevalence of mistreatment but also its negative consequences for academic achievement and later professional conduct. Although the effects on students' mental health, wellbeing and perceptions about medical education and academic achievements are well known,^5^^,^^7^^,^^12^^,^^16^^,^^17^^,^^18^^,^^19^^,^^20^ studies addressing the perceived severity and recurrence of mistreatment and the association with students' perceptions about their medical course are scarce. Most of the studies have been conducted in the US, and no data are available for Brazil.

Medical courses in Brazil have a minimum duration of six years. Basic science disciplines are usually provided in the first two years, and disciplines focusing on training for general medical practice and medical specialties in the third and fourth years. Internship, i.e. the mandatory clinical training period, takes place in the last two years, with activities within healthcare services. In Brazil, students start medical education earlier; medical education may be the first undergraduate course and may immediately follow the secondary school cycle. Thus, medical education can start as early as the ages of 17 or 18 years, with no previous university or college experience. Exposure to different forms of mistreatment within the academic environment at such an early age can have an even more pronounced negative impact, especially in cases that are recurrent or that students consider important. Our hypothesis was that mistreatment that was perceived as severe or recurrent was associated with a negative impact on the way in which students perceive medical education and their academic achievements.

## OBJECTIVE

Our aim was to estimate the prevalence of mistreatment that was perceived as severe and recurrent among medical students and to investigate the association with medical students' perceptions about their medical course.

## METHODS

### Study design and participants

The QUARA project (Quality of Relationships in the Academic Environment) was a cross-sectional study undertaken in a Brazilian public medical school in the city of São Paulo from September to December 2013 that evaluated medical students with regard to the following: mistreatment, socioeconomic characteristics, aspects of the medical course, mental health problems before starting the medical course, lifestyle habits, depression, burnout, social support, quality of life, stressful life events and professional conduct.

All the medical students who were formally registered in 2013 in the same medical school (n = 1,072) were invited to participate in the study, through an initial email containing a link to an informed consent statement and the full questionnaire. Weekly reminders followed the initial email invitation. Additional awareness-raising initiatives included face-to-face reminders during lectures by a student reference group, Facebook posts and distribution of flyers.

### Procedures

To facilitate data collection, an online questionnaire was created using REDCap (Research Electronic Data Capture).^21^ A pilot study was conducted (n = 10) before the beginning of data collection in order to test the procedures and identify unclear questions. Authorization for translation and use of the questionnaire Perception of Medical Students on their Learning Environment^4^ was obtained from its author.

The ethics committee of the Medical School of the University of São Paulo (FMUSP) approved this research. Privacy and confidentiality were guaranteed for all participants, and all students signed an informed consent form before participation (Ref 345.993, July 31, 2013).

### Measurements

#### Mistreatment

The questions about mistreatment were derived from the questionnaire Perception of Medical Students on their Learning Environment^4^ and were translated by two independent translators following three steps: 1) translation into Portuguese, 2) back-translation and 3) group discussion to establish the final version. The validity of the questionnaire was not assessed.

This questionnaire addresses the following types of mistreatment by different perpetrators (professors, students, residents, preceptors/supervisors, attending physicians, nurses, other healthcare professionals, patients or their families and others): shouting/yelling, depreciation/humiliation, task assignment with punitive purposes, derogatory comments about the career, racial/religious discrimination, threat of injury, threat of physical harm, sexual harassment and discrimination and physical violence (slap, push, kick or hit). The responses are 1 (never), 2 (rarely; 1-2 times), 3 (sometimes; 3-4 times) and 4 (often; 5 times or more). For this paper, we took exposure to any type of mistreatment to be a binary variable (0: never/rarely/sometimes; or 1: often, 5 times or more). If a student answered "five times or more" for at least one type of mistreatment, this was considered to be recurrent mistreatment.

An additional question assesses how much each of the mistreatments bothered the student. The responses include the following options: 0 (does not apply; was not a victim), 1 (not at all), 2 (a little) and 3 (a lot). For the purpose of our analysis, a binary variable expressing the importance attributed by the students to the mistreatment suffered was recoded as 0 (was not a victim/not at all, a little) or 1 (a lot). If a student answered "bothered a lot" for at least one type of mistreatment, this was considered to be mistreatment perceived as severe by the student. This variable expresses students' perception, and does not take any external parameter of gravity into consideration.

### Perception of medical course

Four questions addressed the students' perceptions concerning their medical course: 1) Are you satisfied with your professional choice? a) yes or b) no/I don't know; 2) Have you ever considered dropping out of this course? a) no or b) yes, I did in the past/yes, I still think about it; 3) Do you think your academic achievement is excellent, good, fair or poor? a) excellent/good or b) fair/poor; 4) Do you feel overloaded by the activities you perform as part of your course? a) yes or b) no.

### Statistical analysis

All the data analyses were performed using Stata 13.0. The prevalence of exposure to recurrent mistreatment or mistreatment perceived as severe was calculated. The associations that recurrent mistreatment and mistreatment perceived as severe presented in relation to the perception of overload, dissatisfaction with the medical course, willingness to drop out and perception of poor academic achievement were calculated through Poisson regression analysis after adjusting for sex, age, skin color, whether student was admitted to course through the social inclusion policy and school cycle. Poisson regression was the statistical method chosen because this method enables calculation of prevalence ratios and gives better estimates when dealing with high-prevalence outcomes.

The prevalence and prevalence ratios were estimated after ranking adjustments and post-stratification weighting by considering the distribution of all the students in the medical school according to gender and class for the whole sample and according to school cycle. Confidence intervals (95%) were calculated for all point estimates. The chi-square test was used to test for linear trend according to school cycle.

## RESULTS

Out of the 1,072 students invited, 338 (31.5%) agreed to participate in the survey. Of these, 317 students completed the interview. Approximately 50% of the respondents were female. Most participants were 23 years old or older, and the mean age was 22.4 years (standard deviation, SD = 2.8). White was the skin color most reported. Nearly 45% of the sample was in the basic science years of medical education, and only 22% were in the clinical cycle (internship) (Table 1).

**Table 1:**
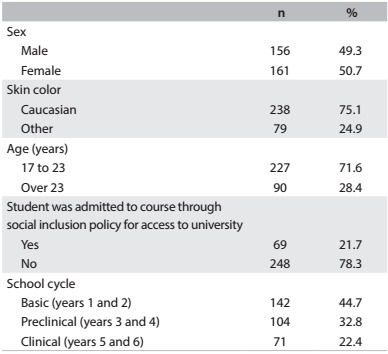
Characteristics of the participants (n = 317). São Paulo, Brazil, 2013

The overall prevalence of mistreatment among medical students during their training years was extremely high (92.3%) (Table 2). Recurrent mistreatment was reported by 30.1% of the participants, and most students (64.2%) reported having been exposed to mistreatment that they perceived as severe. The exposure to overall, recurrent and severe mistreatment increased over the duration of the course, with higher prevalence during the clinical years. A significant linear trend was found for severe mistreatment. This probably expresses the cumulative dimension of our measurement, since we asked about occurrences of mistreatment over the duration of the course.

**Table 2:**
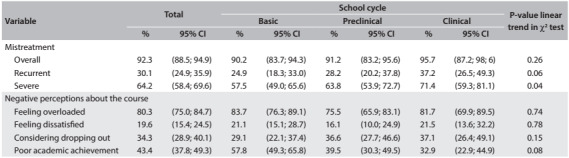
Exposure to mistreatment and negative perceptions about the medical course in São Paulo, Brazil, 2013

CI = confidence interval.

A negative perception of the medical course was common in our sample: 80% of the students felt overloaded, nearly 20% reported feeling dissatisfied and nearly 35% had considered dropping out of their medical course (Table 2). Basic year students presented negative perceptions about the course more frequently. Clinical year students reported having considered dropping out of the course more frequently. No linear trend was found.

Being exposed to recurrent mistreatment (five times or more) was not associated with any of the indicators of negative perception about the course (Table 3). However, being exposed to mistreatment that was perceived as severe was associated with higher prevalence of perceived overload and considering dropping out of the course, in both the crude and the adjusted models. Students who were exposed to mistreatment that they perceived as severe felt more overloaded (prevalence ratio, PR = 1.17; 95% confidence interval, CI: 1.02; 1.36) and had considered dropping out of the course more frequently than those not exposed (PR = 2.15; 95% CI: 1.37; 3.36). The association between mistreatment perceived as severe and dissatisfaction with the course (PR = 1.67; 95% CI: 0.94; 2.99) reached a borderline significant level (P = 0.07).

**Table 3:**
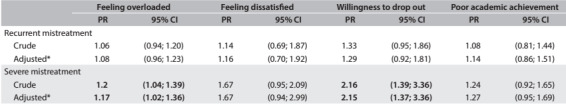
Association between recurrent or severe mistreatment and negative perception about the medical course in São Paulo, Brazil, 2013

PR = Poisson logistic regression. Model adjusted for gender, age, skin color, whether student was admitted to course through the social inclusion policy and school cycle; CI = confidence interval.

## DISCUSSION

This was the first study to take a systematic approach towards investigating exposure to recurrent mistreatment and mistreatment perceived as severe and their associations with students' perceptions about their medical course in a Brazilian medical school. Students in their first to sixth year were enrolled, and data were collected using an online system that guaranteed full anonymity and confidentiality. We used a structured questionnaire to measure mistreatment, classified according to frequency and perceived severity.

Participation was not mandatory, and we had a fair response rate (31.5%). Our final sample represented 29.45% of the total original population (n = 317). Low response rates seem to be a problem common to many web-based surveys.^22^^,^^23^ In an online survey concerning negative experiences during medical education at the University of Göttingen, in Germany, a similar response rate of 32% was found.^10^ Since participation in our survey was not mandatory, the possibility of selection bias also has to be borne in mind: this might have resulted either in overestimation of our prevalence estimates (since voluntary surveys attract the attention of those more interested in or affected by the topic) or even in underestimation (because the response rates were lower among the students in their clinical years/internship, when occurrences of mistreatment tend to be more frequent).

It is impossible to know whether those who participated in this study were more or less exposed to mistreatment, but our results are consistent with those reported by other studies. Furthermore, our final sample had more women and fewer clinical-year students than a reference population. To address this imbalance, we used post-stratification weighting for sex and school year, with adjustments for all point and interval estimates. It is important to note that this was a cross-sectional study, and it was not possible to ascertain whether the mistreatment occurred prior to the development of negative perception about the medical course.

Exposure to mistreatment that was considered severe was positively associated with perceived overload and willingness to drop out of the course. The exposed students reported feeling overwhelmed more frequently than did those who were not exposed, and expressed willingness to drop out of the course more frequently than did those who were not exposed, even after adjusting for potential confounders. These results support our hypothesis that there would be an independent association between exposure to mistreatment that was perceived as severe and negative perceptions about the course.

### Prevalence of exposure to violence

According to our results, nearly every student suffered at least one form of mistreatment during the medical course. The overall prevalence was extremely high, making it evident that this phenomenon is present in the everyday life of students and plays an important role in the academic environment in medical school, within all school cycles. Even when only the cases that were judged to be severe and those that were recurrent (five times or more during the course) were taken into consideration, the prevalence was high. According to our results, exposure to mistreatment became higher as the course progressed, thus reflecting the cumulative dimension of our measurement. We were unable to ascertain the time when mistreatment occurred with any certainty, since our questionnaire asked about experiences at any time during the course. However, the high prevalence found during the basic years reflects exposure during the first two years of medical training, a period when students might be especially vulnerable to the consequences of mistreatment, given that they enter the medical course early in life in Brazil, immediately following high school and with no previous experience of college or university dynamics.

Similar high figures have previously been described in the United States,^1^^,^^5^ Chile,^3^ Nigeria^12^ and Germany.^10^ According to Baldwin et al.,^1^ 96.5% of the students in the fourth and fifth years in 10 medical schools in the United States reported having had exposure to at least one type of mistreatment (assault, harassment or mistreatment) during the course. More recent results reported by Frank et al.^5^ have indicated that the prevalence remains high: 84% of the students reported having been belittled during the course in a study conducted in 16 US medical schools. In Nigeria, Chile and Germany, the overall prevalences were, respectively, 98.5%, 91.5% and 88%. A recent meta-analysis^24^ reported that the pooled prevalence of harassment and discrimination among medical students was of the order of 59.6% (95% CI: 49.2%; 68%) and that the pooled prevalence of verbal harassment was 68.8% (95% CI: 56.6%; 80.9%).

Lower prevalence of mistreatment has been reported in relation to episodes of bullying characterized by recurrent violence. In Saudi Arabia, Alzahrani^9^ reported that the prevalence of bullying was 28% among medical students, whereas in Pakistan, the prevalence was 52% among sixth-year medical students.^6^ In Colombia, Paredes et al.^8^ reported that the prevalence of bullying was 19.7%. In our study, 30.1% of the students reported a pattern of repeated exposure to mistreatment (> 5 episodes) during the course.

It is noteworthy that such high prevalence persists and is widespread in so many different countries and scenarios. In the early 1990s, following the study published by Sheehan et al.,^15^ the high prevalence of assault, abuse and harassment in medical education was brought to light, as was the existence of a cycle of abusive practices that had been incorporated as a "necessary part" of medical education. According to Fried et al.,^14^ the high frequency of abuse, mistreatment and other forms of aggression within medical courses demonstrates the existence of a strongly hierarchical medical culture that permeates the relationship between teachers and students, thus perpetuating situations of maltreatment as "rites of passage". According to our data, this pattern of relationship is present starting from the first years of medical education and persists throughout the course. This culture is, according to Kay,^25^ a problem that goes beyond the undergraduate years and persists over time in different spaces of medical training, where attending physicians, supervisors, residents and students reproduce the abuse and mistreatment suffered during the formative years in a cycle that feeds itself.

### Consequences of mistreatment on the way students perceive medical education

The existence of a pattern of relationships based on humiliation and psychological violence, both between students and in teaching relationships, may negatively affect the way in which students perceive medical education and professional choice.^5^^,^^16^^,^^20^ Our results brought to light that a high proportion of students have negative perceptions about the course, especially during the basic cycle, in which the figures were higher for all indicators except for considering dropping out of the course, which was seen more frequently during the clinical cycle. This may be a reflection of the cumulative dimension of this specific measurement, since we asked whether the students had ever considered dropping out the course. Feeling overloaded, being dissatisfied and having poor academic achievement were all related to the students' experiences and thus these characteristics closely reflect the students' actual experience with the course. In this sense, it is quite surprising that in the basic cycle, the figures were of such high magnitude. This can be explained by the early age at which the students started their training in such a hard and demanding course as medical education, with the need to deal with academic stress, the strong hierarchy mentioned previously and occurrences of mistreatment.

It should be noted that according to our results, episodes of mistreatment are not only highly prevalent but also recurrent and are judged to be severe by the students. Our results demonstrate that the mistreatments that the students considered to be severe were associated with negative impressions about the course. Feeling overwhelmed and considering dropping out of the course were seen more frequently among those who judged the mistreatment to be severe, even after adjustment for potential confounders.

Rosenberg^16^ described the negative consequences of mistreatment on academic achievement and subsequent professional conduct in the 1980s. According to these authors, abuse is related to poorer learning, lower self-esteem and lower quality of patient care. Recent studies have indicated that students who have been the victims of abuse, maltreatment or other forms of aggression are more dissatisfied with their career choice, are more likely to consider dropping out and report poorer relationships with teachers.^5^ According to Timm,^11^ students who have been exposed to bullying or harassment find it harder to concentrate and are less satisfied with their career choice. Mistreatment also has consequences for students' mental health. Those who are exposed feel more stressed and depressed, have low self-esteem and are more likely to consume alcohol and binge-drink.^5^^,^^7^^,^^12^^,^^17^^,^^18^^,^^19^

The different sources of stress to which medical students are exposed during their medical training are well known.^18^^,^^26^^,^^27^ Medical education is hard to access and difficult to sustain, given the large amount of time dedicated to academic activities and the amount of suffering and distress that students need to cope with on a daily basis. Overwhelming activities, along with contact with disease and death early in life, are among the factors that explain the high prevalence of depression and burn-out and the perception of poor quality of life that are commonly reported in medical student surveys.^28^^,^^29^^,^^30^^,^^31^^,^^32^^,^^33^^,^^34^

According to Dyrbey et al.,^35^ unprofessional conduct and less altruistic professional values are more common among students with burn-out, thus suggesting that distress during medical education may compromise quality of care. Similar results were found in a study involving 1098 medical students in the United States,^36^ in which distress and lack of wellbeing were shown to present connections with lack of empathy among medical students. In the same study, perceptions of personal accomplishment and high quality of life were both associated with higher empathy.

Despite the fact that mistreatment has been found to be highly prevalent in different countries and during different time periods, this study provided the first systematic approach towards this topic in a Brazilian medical school. It should be noted that this is an opportune time to bring this discussion to the forefront in this country, because the new national curriculum guidelines that were recently approved state that medical schools should be able to provide training for doctors to practice "general, humanistic, critical, reflective and ethical medicine (...) with social responsibility and commitment to the defense of citizenship [and] human dignity".^37^ Considering the negative impact of mistreatment on mental health and wellbeing, and the way in which students perceive their course and academic achievements,^5^^,^^7^^,^^12^^,^^16^^,^^17^^,^^18^^,^^19^^,^^20^ occurrences of mistreatment during medical training have high potential to compromise quality of care and the way in which students approach their patients and their suffering. Mistreatment represents an additional source of stress for medical students that should be seriously taken into consideration by medical schools.

## CONCLUSION

Mistreatment is highly prevalent within medical education in Brazil, Mistreatment that is perceived as severe by students has a negative impact on the way in which they perceive their course. In our study, however, recurrent mistreatment per se was not associated with negative perceptions about the course or poor academic achievement.

Our results suggest that the subjective dimension of mistreatment (i.e. the students' perceptions regarding its severity) is more important than recurrence, in considering the impact on the way in which students perceive their course, and this should be taken into considered in future investigations.
